# The Four-Dimensional Symptom Questionnaire (4DSQ): a validation study of a multidimensional self-report questionnaire to assess distress, depression, anxiety and somatization

**DOI:** 10.1186/1471-244X-6-34

**Published:** 2006-08-22

**Authors:** Berend Terluin, Harm WJ van Marwijk, Herman J Adèr, Henrica CW de Vet, Brenda WJH Penninx, Marleen LM Hermens, Christine A van Boeijen, Anton JLM van Balkom, Jac JL van der Klink, Wim AB Stalman

**Affiliations:** 1 Department of General Practice, EMGO-Institute, VU University Medical Centre, Van der Boechorststraat 7, 1081 BT Amsterdam, The Netherlands; 2 Department of Clinical Epidemiology and Biostatistics, VU University Medical Centre, Van der Boechorststraat 7, 1081 BT Amsterdam, The Netherlands; 3 EMGO-Institute, VU University Medical Centre, Van der Boechorststraat 7, 1081 BT Amsterdam, The Netherlands; 4 Department of Psychiatry, EMGO-Institute, VU University Medical Centre, Van der Boechorststraat 7, 1081 BT Amsterdam, The Netherlands; 5 Academic Medical Centre, Coronel Institute, University of Amsterdam, Meibergdreef 15, 1105 AZ Amsterdam, The Netherlands

## Abstract

**Background:**

The Four-Dimensional Symptom Questionnaire (4DSQ) is a self-report questionnaire that has been developed in primary care to distinguish non-specific general distress from depression, anxiety and somatization. The purpose of this paper is to evaluate its criterion and construct validity.

**Methods:**

Data from 10 different primary care studies have been used. Criterion validity was assessed by comparing the 4DSQ scores with clinical diagnoses, the GPs' diagnosis of any psychosocial problem for Distress, standardised psychiatric diagnoses for Depression and Anxiety, and GPs' suspicion of somatization for Somatization. ROC analyses and logistic regression analyses were used to examine the associations. Construct validity was evaluated by investigating the inter-correlations between the scales, the factorial structure, the associations with other symptom questionnaires, and the associations with stress, personality and social functioning. The factorial structure of the 4DSQ was assessed through confirmatory factor analysis (CFA). The associations with other questionnaires were assessed with Pearson correlations and regression analyses.

**Results:**

Regarding criterion validity, the Distress scale was associated with any psychosocial diagnosis (area under the ROC curve [AUC] 0.79), the Depression scale was associated with major depression (AUC = 0.83), the Anxiety scale was associated with anxiety disorder (AUC = 0.66), and the Somatization scale was associated with the GPs' suspicion of somatization (AUC = 0.65). Regarding the construct validity, the 4DSQ scales appeared to have considerable inter-correlations (r = 0.35-0.71). However, 30–40% of the variance of each scale was unique for that scale. CFA confirmed the 4-factor structure with a comparative fit index (CFI) of 0.92. The 4DSQ scales correlated with most other questionnaires measuring corresponding constructs. However, the 4DSQ Distress scale appeared to correlate with some other depression scales more than the 4DSQ Depression scale. Measures of stress (i.e. life events, psychosocial problems, and work stress) were mainly associated with Distress, while Distress, in turn, was mainly associated with psychosocial dysfunctioning, including sick leave.

**Conclusion:**

The 4DSQ seems to be a valid self-report questionnaire to measure distress, depression, anxiety and somatization in primary care patients. The 4DSQ Distress scale appears to measure the most general, most common, expression of psychological problems.

## Background

The Four-Dimensional Symptom Questionnaire (4DSQ) is a Dutch self-report questionnaire designed to assess common psychological symptoms in primary care patients [1]. The 4DSQ's special feature is its ability to distinguish general distress from depression, anxiety and somatization. The aim of this paper is to investigate the 4DSQ's validity. Validity refers to the degree to which an instrument measures what it purports to measure. Two kinds of validity will be presented: criterion validity, the extent to which the 4DSQ scores are related to criterion measures, and construct validity, the extent to which the 4DSQ scores are in accordance with theoretical considerations [2]. Other psychometric properties (i.e. reliability, precision, smallest detectable change, responsiveness and respondent burden) will be presented in additional files to this paper (see Additional files 1, 2, 3). First, we shall describe the 4DSQ and its conceptual background.

### The Four-Dimensional Symptom Questionnaire (4DSQ)

The 4DSQ is a self-report questionnaire comprising 50 items distributed over four scales. The items are worded as questions similar to those that can be asked in everyday primary care practice. The reference period is "the past week". For example, item 26 reads "During the past week, did you feel easily irritated?". The 4DSQ does not contain any positive affect questions, nor any other "reversed" worded questions. The response categories are also worded as normal answers to clinical questions: "no", "sometimes", "regularly", "often", "very often or constantly". In order to arrive at scale scores, the responses are scored as 0 for "no", 1 for "sometimes" and 2 for the other response categories, and the item scores are summated to scale scores. The Distress scale contains 16 items and has a score range of 0–32, the Depression scale contains 6 items and has a range of 0–12, the Anxiety scale contains 12 items and has a range of 0–24, and the Somatization scale contains 16 items and has a range of 0–32 (Table 1). The 4DSQ is free for non-commercial use in health care and research and it is available as a Dutch and an English version [3]. The English version was obtained through a procedure of translation into English and back-translation into Dutch. The English translation was adjusted to obtain the best equivalence with the original Dutch 4DSQ.

**Table 1:** Four-Dimensional Symptom Questionnaire (4DSQ): items, scale, and factor loadings (confirmatory factor analysis, test-set)

*During the past week, did you suffer from:*			
1. dizziness or feeling light-headed?	Som		0.51
2. painful muscles?	Som		0.44
3. fainting?	Som		0.21
4. neck pain?	Som		0.40
5. back pain?	Som		0.37
6. excessive perspiration?	Som		0.42
7. palpitations?	Som		0.61
8. headache?	Som		0.30
9. a bloated feeling in the abdomen?	Som		0.53
10. blurred vision or spots in front of your eyes?	Som		0.51
11. shortness of breath?	Som		0.63
12. nausea or an upset stomach?	Som		0.47
13. pain in the abdomen or stomach area?	Som		0.46
14. tingling in the fingers?	Som		0.50
15. pressure or a tight feeling in the chest?	Som		0.70
16. pain in the chest?	Som		0.65
17. feeling down or depressed?	Dis		0.66
18. sudden shock for no reason?	Anx		0.52
19. worry?	Dis		0.45
20. disturbed sleep?	Dis		0.39
21. indefinable feelings of fear?	Anx		0.74
22. listlessness?	Dis		0.66
23. trembling when with other people?	Anx		0.57
24. anxiety or panic attacks?	Anx		0.79
*During the past week, did you feel:*			
25. tense?	Dis		0.50
26. easily irritated?	Dis		0.53
27. frightened?	Anx		0.79
28. that everything is meaningless?	Dep		0.75
29. that you just can't do anything anymore?	Dis		0.76
30. that life is not worth while?	Dep		0.86
31. that you can no longer take any interest in the people and things around you?	Dis		0.71
32. that you can't cope anymore?	Dis		0.80
33. that you would be better off if you were dead?	Dep		0.79
34. that you can't enjoy anything anymore?	Dep		0.70
35. that there is no escape from your situation?	Dep		0.73
36. that you can't face it anymore?	Dis		0.79
*During the past week, did you:*			
37. no longer feel like doing anything?	Dis		0.79
38. have difficulty in thinking clearly?	Dis		0.63
39. have difficulty in getting to sleep?	Dis		0.41
40. have any fear of going out of the house alone?	Anx		0.65
*During the past week:*			
41. did you easily become emotional?	Dis		0.50
42. were you afraid of anything when there was really no need for you to be afraid? (for instance animals, heights, small rooms)	Anx		0.64
43. were you afraid to travel on busses, trains or trams?	Anx		0.59
44. were you afraid of becoming embarrassed when with other people?	Anx		0.53
45. did you ever feel as if you were being threatened by unknown danger?	Anx		0.54
46. did you ever think "If only I was dead"?	Dep		0.77
47. did you ever have fleeting images of any upsetting event(s) that you have experienced?	Dis		0.40
48. did you ever have to do your best to put aside thoughts about any upsetting event(s)?	Dis		0.45
49. did you have to avoid certain places because they frightened you?	Anx		0.63
50. did you have to repeat some actions a number of times before you could do something else?	Anx		0.42

Dis = Distress, Dep = Depression, Anx = Anxiety, Som = Somatization

### Conceptual background

The 4DSQ is grounded in our study of the clinical characteristics of patients with a "nervous breakdown" presenting in general practice [4]. Dutch general practitioners (GPs) use the label "nervous breakdown" (NB, or "overstressed"/"overburdened", in Dutch "overspanning") as a proper diagnosis to denote a syndrome that is associated with overwhelming life stress to the extent that the patient cannot cope anymore [5,6]. In our study, it appeared that NB patients typically showed a syndrome of non-specific symptoms that we have called "distress". We have studied the distribution of common psychological symptoms in a large random sample of general practice patients and discovered that most of the variance of these symptoms could be described by just four symptom dimensions: distress, depression, anxiety and somatization [4]. In some patients (notably NB patients) distress was more or less a stand alone problem, whereas in other patients distress was combined with depression, anxiety and/or somatization. The distinction between distress on the one hand, and depression, anxiety and somatization on the other hand, implies a distinction between "normal" reactions to stress and "abnormal" – psychiatric – disorders.

#### Distress

Characteristic distress symptoms are worry, irritability, tension, listlessness, poor concentration, sleeping problems and demoralisation. Mild distress states, which do not interfere much with normal social functioning, can be considered to be part of normal daily life. However, severe distress states (as in NB) force a patient to give up and withdraw from major social roles, especially the occupational role. We conceptualize distress as the direct manifestation of the effort people must exert to maintain their psychosocial homeostasis and social functioning when confronted with taxing life stress [7]. The source of the stress may be anything that has the ability to threaten one's bio-psycho-social homeostasis (e.g. work load, conflicts, life events, traumatic experiences, psychosocial difficulties, grief, somatic disease, or psychiatric disorder).

#### Depression and anxiety

Because distress symptoms virtually always accompany mood and anxiety disorders, it seems difficult to differentiate distress from depression and anxiety. However, when we try to separate distress from depression and anxiety, what is left must be the very core of these psychiatric disorders. When distress is separated from depression we are left with anhedonia and depressive thoughts. These symptoms are considered to represent the core symptomatology of major depression [8,9]. When we separate distress from anxiety, we are left with irrational fears, anticipation anxiety and avoidance behaviour. These symptoms are characteristic of the various anxiety disorders [10]. Whereas distress is primarily a manifestation of a stress-coping problem, depression and anxiety are triggered or aggravated by still poorly understood dysfunctions of mood and anxiety regulation systems [11].

#### Somatization

Somatization is a tendency to experience medically unexplained somatic symptoms, to attribute them to physical illness, and to seek medical help for them [12]. Various mechanisms may contribute to somatization, including sensitisation of the brain to bodily sensations [13], physiological abnormalities in the nervous and endocrine systems [14], heightened awareness of bodily sensations [15], and inappropriate illness beliefs and sickness behaviour [16]. Experiencing one or just a few medically unexplained symptoms (e.g. dizziness or upset stomach) is common in "normal" people under stressful circumstances [13,17]. However, experiencing many unexplained symptoms from different organ systems (e.g. dizziness ánd upset stomach ánd palpitations ánd muscular aches) implies somatization as described above [16].

A non-specific general distress factor has been described in the literature before. However, there seems to be little consensus about the exact relationship between distress and anxiety/depression. Goldberg et al. described distress as a general factor of depression and anxiety, representing the overall severity of the disorder [18]. Dohrenwend et al. described a general distress factor as the common factor underlying many psychiatric screening lists [19]. Clark and Watson introduced the tripartite model in which general distress is viewed as a personality trait – "negative affectivity" – that is underlying depressive and anxiety disorders as a non-specific predisposition [20].

In our four-dimensional model of psychological symptoms, distress is not merely a (common) part of anxiety and depression but, instead, we see distress as a fourth dimension of psychopathology besides depression, anxiety and somatization. Distress is the most basic, most general, most "normal" expression of psychological problems. In principle, distress is independent from depression, anxiety and somatization. However, in practice, the dimensions are correlated, presumably because the strain that produces distress, may also trigger disturbances underlying depression, anxiety or somatization in vulnerable people.

## Methods

We have used 10 different datasets, which are described in Appendix 1 (Table 2). In order to evaluate the criterion validity of the 4DSQ scales we studied the association between the 4DSQ scores and relevant clinical diagnoses, GPs' diagnoses for Distress and Somatization, and standardised psychiatric diagnoses for Depression and Anxiety. To evaluate the construct validity of the 4DSQ scales we examined the inter-correlations between the scales, the factor structure, the associations with other symptom questionnaires, and the associations with relevant other constructs like stress factors, personality and social functioning. We hypothesised that distress was more strongly associated with stress and social functioning than depression, anxiety and somatization. Because personality determines to a certain extent how a person reacts to stress, we hypothesised that distress was also more strongly associated with personality than depression, anxiety and somatization. All analyses, except for the factor analysis, were performed using SPSS 12.0.

**Table 2:** Overview of the study samples used in this paper

Study	Sample	n	% women	Mean age (SD)
A	general practice patients	2127	68	38.5 (11.5)
B	employees responding to a health survey	3852	9	43.9 (8.1)
C	employees with adjustment disorder	280	34	41.9 (8.1)
D	distressed general practice patients	55	53	40.4 (10.6)
E	anxious general practice patients	237	62	37.8 (12.4)
F	GP patients with minor or mild-major depression	178	74	44.8 (15.7)
G	social work clients	77	66	35.0 (8.3)
H	physiotherapy patients	382	61	40.7 (12.5)
I	GP patients with psychological problems	86	66	40.2 (10.0)
J	GP patients with psychological problems	129	58	42.5 (12.7)

### Descriptives

To provide more detailed information about the samples, we have calculated mean scores and standard deviations of the 4DSQ scales in the various study samples. The significance of the differences between samples was tested using one-way analysis of variance (ANOVA) with Bonferroni post hoc multiple comparisons (p < 0.05). Comparing the mean scores across samples provided already an aspect of construct validity. For example, it was to be expected that primary care patients would have higher scores than employees, and samples with high prevalences of depressive disorders (study D) or anxiety disorders (study E) would have the highest scores for Depression and Anxiety respectively. Moreover, this information provided an idea about the interpretability and applicability of the 4DSQ in different populations.

### Criterion validity: associations with clinical diagnoses

To examine criterion validity we compared the 4DSQ Distress and Somatization scores with GPs' diagnoses, and the 4DSQ Depression and Anxiety scores with standardised psychiatric diagnoses. In every instance, the differences in 4DSQ scores between the diagnostic groups (e.g. major depression vs. no major depression) were examined using one-way ANOVA. To investigate whether the 4DSQ score could "detect" the diagnosis and would perform better than the other 4DSQ scales, we calculated ROC curves for the 4DSQ scales. Differences in areas under the ROC-curve (AUCs) were tested using the method outlined by Hanley and McNiel [21]. We performed also a logistic regression analysis with the diagnosis as dependent variable and the 4DSQ scores as independent variables, building a parsimonious model by stepwise backward elimination of non-significant "predictors" (p > 0.05).

#### Distress

Conceptually, as mentioned above, distress represents the most general expression of any psychological problem. Since a true valid and reliable gold standard for distress is not available, we adopted "any psychosocial diagnosis/reason for encounter" as established by the GP as the "criterion" for the 4DSQ Distress score. This criterion should represent a reasonable indicator of distress, with relevance for primary care. Given the unknown reliability of the criterion, any relationship with the 4DSQ Distress score can be interpreted as supporting validity of the 4DSQ Distress scale. In study A 2,127 consecutive primary care patients filled in a 4DSQ while the GPs recorded their diagnoses/reasons for encounter. These diagnoses/reasons for encounter were later categorised as either "psychosocial" or "somatic" by a researcher who was blinded for the 4DSQ scores. Examples of psychosocial diagnoses are "anxiety disorder", "sleeping problem", "nervousness", and "marriage problem". All diagnoses/reasons for encounter not overtly implying a psychosocial problem were coded as "somatic".

#### Depression

We considered the standardised psychiatric diagnosis of a current major depressive disorder as the "criterion" for the 4DSQ Depression score. In study D 55 GP patients with psychological symptoms were interviewed twice by their GP with an interval of 1–2 days. The diagnosis DSM-IV major depression was made using the Short Depression Interview (SDI) [22]. The patients filled in the 4DSQ at both occasions. In order to increase the power of the analysis, the measurements of both times were combined as if the retests were independent observations.

#### Anxiety

We adopted the standardised psychiatric diagnosis of a current anxiety disorder as the "criterion" for the 4DSQ Anxiety score. In study E 206 GP patients, selected because of a positive result of a screening test for anxiety, were interviewed by a mental health professional using the Structured Clinical Interview for DSM-IV (SCID) [23]. The patients filled in the 4DSQ.

#### Somatization

We adopted the GP's suspicion of a psychosocial background in the case of a somatic diagnosis/reason for encounter as the "criterion" for the 4DSQ Somatization score. This criterion is not meant to be a true gold standard for somatization, but rather a reasonable indicator of somatization (i.e. the presentation of somatic complaints unexplained by physical illness), relevant for primary care. Given the unknown reliability of the criterion, any relationship with the 4DSQ Somatization score can be interpreted as supporting validity of the 4DSQ Somatization scale. In study A the GPs not only recorded their diagnoses/reasons for encounter, but they also rated the background of the diagnosis/reason for encounter on a 5-points somatic-psychosocial scale. Patients with a somatic diagnosis/reason for encounter were categorised into three groups according to the somatic-psychosocial score: "definite somatization" (score 4–5), "possible somatization" (score 2–3), and "no somatization" (score 1). We investigated the association between the 4DSQ scores and the GPs' suspicion of somatization in patients with a somatic diagnosis.

### Construct validity: inter-correlations between the 4DSQ scales

Pearson correlation coefficients were calculated between the 4DSQ scales in three samples: the GP patients of study A, the employees of study B, and a pooled sample of studies C through J. In order to test whether or not each 4DSQ scale had at least some unique variance that was not shared with the other scales, we regressed each of the scales onto the other scales, and calculated the standardised Beta coefficients and the explained proportion of variance (R^2^). Because Cronbach's α is an estimate of the proportion of the total variance of a scale that is error free, and R^2 ^is the proportion of the total variance that the scale shares with the other scales, the difference between α and R^2 ^is an estimate of the unique variance of the scale. Finally, we looked at the scatterplots of pairs of 4DSQ scales for remarkable patterns.

### Construct validity: factorial structure of the 4DSQ

The factorial structure of the 4DSQ was investigated using the data of studies C through J. We used confirmatory factor analysis (CFA) as implemented in EQS, a program for structural equation modelling [24]. Because some of the variables showed considerable kurtosis, we used elliptical reweighted least squares (ERLS) estimation. This approximation corrects for violations of the Gaussian kurtosis assumption. Because EQS cannot run with missing values, these were imputed in SPSS 12.0 with the expectation-maximisation procedure. The data set was randomly split into two sets of similar size. The first set was used to explore the data and develop the models; the second set was used to test them. We indicate these sets with "exploration set" and "test set", respectively.

First, a 4-factor model was fitted, the factors being in accordance with the 4DSQ scales. The relationships between the factors were examined by exploring various possibilities of one factor "explaining" another factor, or two factors simply showing an association. The comparative fit index (CFI) was used to evaluate the fit of the model. A CFI of ≥ 0.90 is generally considered as an indication of an adequate fit of the model to the data [25]. In order to investigate whether the 4-factor model could be further improved, we tried some adjustments of the 4-factor model as well as a 5-factor model in which items that were cross-loading on the distress and depression factors were handled as a fifth factor.

### Construct validity: correlations with other symptom questionnaires

The correlations between the 4DSQ scales and other psychological symptom questionnaires can be interpreted as indications of *convergent validity *(when two scales purported to measure similar phenomena show a relatively high correlation) or *divergent validity *(when two scales purported to measure different phenomena show a relatively low correlation). However, since psychological symptoms tend to correlate with each other [26], finding significant positive correlations is little informative. Therefore, we performed linear regression analyses with the comparison questionnaires as dependent variables and the 4DSQ scales as independent variables in order to partial out the relative contributions of the 4DSQ scales to the "explanation" of the comparison questionnaires. Furthermore, we determined the extent to which the comparison questionnaires were covered by the 4DSQ scales by comparing the explained variance (R^2^) with the total error-free variance (Cronbach's α) of the comparison questionnaires.

We used data from studies C, D, F and H to compare the 4DSQ scales with other questionnaires measuring distress, depression, anxiety or somatization. We used the 30-item General Health Questionnaire (GHQ) [27,28] and the Maastricht Questionnaire (MQ) [29] from study D to compare with the 4DSQ Distress scale. The MQ measures "vital exhaustion", a construct that resembles general distress [30]. We used the Symptom Checklist (SCL-90) Depression scale [31,32] from study C, the Hospital Anxiety and Depression Scale (HADS) Depression scale [33,34] and the Self-rating Depression Scale of Zung (SDS) [35,36] from study D, and the Beck Depression Inventory (BDI) [37,38] from study F to compare with the 4DSQ Depression scale. We used the SCL-90 Anxiety and Agoraphobia scales from study C, the HADS Anxiety scale from study D, the Beck Anxiety Inventory (BAI) [39] from study F, and the State-Trait Anxiety Inventory (STAI) State scale [40,41] from study H to compare with the 4DSQ Anxiety scale. Finally, we used the SCL-90 Somatization scale from study C to compare with the 4DSQ Somatization scale.

### Construct validity: associations with stress, personality and social functioning

Stress-related measures were recorded in studies A, B, and G. Studies A and G used a checklist of life events experienced in the past 12 months and a checklist of psychosocial problems experienced in the past 6 months [42], yielding a total number of events/problems by simple summation. In study B work stress was measured with the Job Content Questionnaire (JCQ) scales Psychological job demands, Decision latitude, and Social support from co-workers and supervisors [43].

Personality measures were recorded in studies A, B, C, F, G, and H: two different neuroticism scales, mastery, and trait anxiety. Studies A and G used a Dutch Neuroticism questionnaire based on the work of Eysenck [44], as used by Ormel [45]. Study F included the Neuroticism scale of the NEO Five Factor Inventory (NEO-FFI) [46], studies B and C a measure of mastery [47], and study H a measure of trait anxiety [41].

Social functioning measures were recorded in studies A, F and G. Studies A and G used a simple 5-item questionnaire, inquiring after limitations in social functioning experienced in the following domains: work, household, family, social contacts, and leisure time. The questions were answered on a 5-point scale from "not at all limited" to "very severely limited", and sum scores of the 5 items were used. Studies A and G also used a single question about sick leave with two response options: yes and no. In study F we used the physical and mental component scores of the Short Form Health Survey (SF-36) [48].

The relationships between the 4DSQ scales and the measures of stress, personality and social functioning (except sick leave) were investigated by means of linear regression in which the latter variables were regressed onto the 4DSQ scales. Social (dys)functioning and disability can be considered to be consequences of symptoms such as fatigue, irritability, agitation, poor concentration and sleeping problems. Therefore, it seems appropriate to try to "predict" social (dys)functioning from the 4DSQ scores. However, in the case of stress and personality, the relationship is probably reversed (i.e. stress is assumed to cause psychological symptoms), whereas personality – being related to vulnerability and coping style – is assumed to modify the relationship between stress and symptoms. Thus, using linear regression is like "retrospectively explaining" the relationships between stress and personality, and psychological symptoms. Yet, we have chosen this approach because it is an elegant way of studying the relative effects of stress and personality on the different 4DSQ dimensions. The overall strength of the relationship was expressed in the value of R^2^, the variance of the dependent variable "explained" by the 4DSQ scales, while the overlap between the 4DSQ dimensions was partialled out to reveal the relative effects on the different 4DSQ scales. For the analysis of sick leave logistic regression was used with sick leave as the dependent variable and the 4DSQ scales as the independent variables.

## Results

### Descriptives

Table 3 presents the mean scores and standard deviations of the 4DSQ scales in the various study samples. In all comparisons between samples, the employees (study B) had the lowest mean scores. The general practice patients (study A) had mean 4DSQ scores intermediate between the employees and the selected samples of the studies C through J. The patients from study D had the highest mean Depression and Distress scores. The patients from study E had the highest mean Anxiety and Somatization scores. The adjustment disorder employees (study C) had a mean Distress score almost as high as the study D patients. However, they showed much lower mean scores for Depression and Anxiety, which can be explained by the fact that in study C subjects with major depression or anxiety disorders had been excluded. The social work clients (study G) showed relatively low mean scores for Somatization and Anxiety, compared with the other selected samples. The physiotherapy patients (study H), on the other hand, showed a relatively high mean Somatization score. From the perspective of interpretability these observations all seemed to make sense.

**Table 3:** Means (and standard deviations) of the 4DSQ scores across the study samples. Superscript letters refer to significant (p < 0.05) differences with study letters within the same column (p < 0.05, Bonferroni multiple comparisons)

Study sample	Distress (range 0–32)		Depression (range 0–12)		Anxiety (range 0–24)		Somatization (range 0–32)	
A	9.7^bcdefghij^	(8.5)	1.2^bcdefghij^	(2.6)	2.5^bcdefghij^	(4.0)	8.3^bcdefhij^	(6.2)
B	4.2^acdefghij^	(5.2)	0.4^acdefghij^	(1.2)	0.7^acdefghij^	(1.8)	3.7^acdefghij^	(4.1)
C	22.4^abghj^	(6.7)	3.4^abdef^	(3.2)	5.9^abdeh^	(5.7)	12.9^abegh^	(6.4)
D	24.7^abefghij^	(6.0)	5.9^abcefghij^	(4.0)	8.9^abcdefgij^	(5.6)	14.5^abeg^	(7.9)
E	21.1^abdghj^	(7.1)	4.1^abcdghi^	(3.7)	11.4^abcdfghij^	(6.2)	17.2^abcdfghij^	(6.9)
F	21.0^abdgh^	(7.4)	4.4^abcdghi^	(3.8)	5.9^abdeh^	(5.8)	12.7^abegh^	(7.2)
G	16.9^abcdef^	(8.3)	2.6^abcdefj^	(3.3)	5.1^abdeh^	(4.8)	9.7^bcdefhj^	(6.1)
H	17.9^abcdef^	(8.2)	2.8^abdefj^	(3.3)	7.6^abcefgij^	(6.0)	14.8^abcefgi^	(6.7)
I	20.1^abd^	(8.2)	2.7^abdefj^	(3.6)	5.8^abdeh^	(5.7)	11.9^abehj^	(6.8)
J	18.6^abcde^	(9.1)	3.8^abdghi^	(3.9)	6.3^abdeh^	(6.0)	14.6^abegi^	(7.1)

Note the (very) low mean scores for Depression and Anxiety in the studies A and B. In unselected samples the applicability of the 4DSQ Depression and Anxiety scales appeared to be limited because of relatively low prevalence rates of depressive and anxiety disorders, whereas the Distress and Somatization scales exhibited significant variability.

### Associations with clinical diagnosis

#### Distress

**Figure 1:**
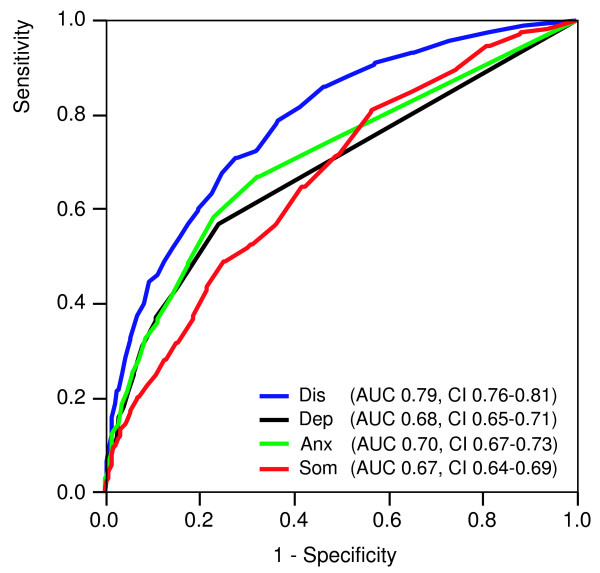
**Receiver operating characteristic (ROC) curves of the 4DSQ scales with respect to detecting a psychosocial diagnosis in general practice patients. **Dis = Distress score, Dep = Depression score, Anx = Anxiety score, Som = Somatization score, AUC = Area Under the Curve, CI = 95% confidence interval.

In study A the GP diagnosis was missing in 90 cases (4%). In 417 of the remaining 2,037 patients (20%) the GP recorded a psychosocial diagnosis/reason for encounter. The psychosocial and somatic groups differed significantly in all four dimensions of the 4DSQ (Table 4). The ROC analysis revealed that all four 4DSQ scales were associated with any psychosocial diagnosis/reason for encounter to some extent (Figure 1), but the Distress score yielded the highest AUC (0.79), being significantly higher than the AUCs of the Depression, Anxiety and Somatization scores (all p-values < 0.001). A Distress cut-off score of ≥ 11 had a sensitivity of 0.71 and a specificity of 0.72 for "detecting" any psychosocial diagnosis/reason for encounter. The final logistic regression model revealed that whether the GP made a psychosocial or a somatic diagnosis was mainly "predicted" by the Distress score (Table 5). With every increase of the Distress score by 1 point (the scale range is 32 points), the odds to receive a psychosocial diagnosis/reason for encounter increased by 13%.

**Table 4:** Differences in 4DSQ scores between patients with a psychosocial and a somatic diagnosis; study A

4DSQ scales	Psychosocial diagnosis (n = 417)		Somatic diagnosis (n = 1620)		F	df	p
				
	Mean	(SD)	Mean	(SD)			
Distress	17.0	(9.0)	7.9	(7.3)	468.6	1/2035	< 0.001
Depression	2.8	(3.7)	0.8	(2.1)	207.4	1/2035	< 0.001
Anxiety	5.3	(5.6)	1.9	(3.3)	253.2	1/2035	< 0.001
Somatization	11.4	(7.0)	7.6	(5.8)	128.3	1/2035	< 0.001

**Table 5:** Logistic regression analysis with psychosocial diagnosis as dependent variable and the 4DSQ scores as independent variables; study A

	Odds ratio^a^	95% CI	p
*Initial model*			
4DSQ Distress	1.14	1.12, 1.17	< 0.001
4DSQ Depression	0.95	0.90, 1.00	0.059
4DSQ Anxiety	1.03	1.00, 1.07	0.073
4DSQ Somatization	0.98	0.96, 1.00	0.109
*Final model*			
4DSQ Distress	1.13	1.11, 1.15	< 0.001

^a ^the odds ratio is associated with one unit of the scale

#### Depression

**Figure 2:**
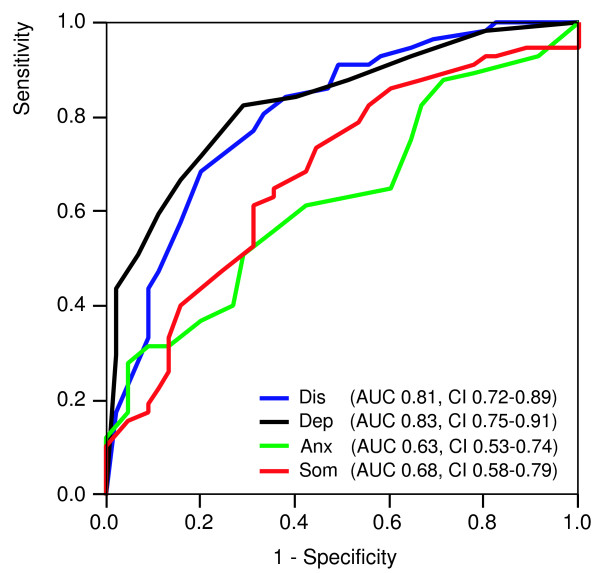
**Receiver operating characteristic (ROC) curves of the 4DSQ scales with respect to detecting a DSM-IV major depression. **Dis = Distress score, Dep = Depression score, Anx = Anxiety score, Som = Somatization score, AUC = Area Under the Curve, CI = 95% confidence interval.

In study D we had 102 instances with both a complete diagnostic assessment and complete results of the 4DSQ. The diagnosis major depression was established in 56%. The mean 4DSQ scores are shown in Table 6. The diagnosis major depression was associated with all four 4DSQ scales. The ROC curves for the 4DSQ scales detecting DSM-IV major depression are shown in Figure 2. The Depression score was the best discriminator between patients with and without a major depression, the AUC of the Depression score (0.83) being significantly larger than the AUCs of the Anxiety and Somatization scores (p-values 0.002 and 0.022 respectively). However, the Distress score performed almost as good, as the difference between the AUCs was not statistically significant (p = 0.555). The Depression score, using a cut-off of ≥ 6, had a sensitivity of 0.72 and a specificity of 0.80 for detecting major depression. Logistic regression analysis showed that the Depression score contributed the most to the prediction of major depression, but curiously, the Somatization score contributed a little to the prediction of major depression in addition (Table 7). For every increase of the Depression score by 1 point (the scale range is 12 points) the odds for having a major depression increased by 46%. Note that the Distress score did not have any additional value with respect to the prediction of major depression with the Depression score already in the equation.

**Table 6:** Differences in 4DSQ scores between patients with and without a major depression; study D

4DSQ scales	Major depression (n = 57)		No major depression (n = 45)		F	df	p
				
	Mean	(SD)	Mean	(SD)			
Distress	27.5	(4.4)	20.7	(6.6)	39.2	1/100	< 0.001
Depression	8.1	(3.8)	3.2	(3.0)	50.2	1/100	< 0.001
Anxiety	9.6	(5.9)	6.8	(4.8)	6.7	1/100	0.011
Somatization	16.4	(7.8)	11.4	(7.5)	10.4	1/100	0.002

**Table 7:** Logistic regression analysis with major depression diagnosis as dependent variable and the 4DSQ scores as independent variables; study D

	Odds ratio^a^	95% CI	p
*Initial model*			
4DSQ Distress	1.07	0.94, 1.22	0.333
4DSQ Depression	1.36	1.12, 1.65	0.002
4DSQ Anxiety	1.00	0.90, 1.12	0.985
4DSQ Somatization	1.09	1.01, 1.16	0.021
*Final model*			
4DSQ Depression	1.46	1.25, 1.69	< 0.001
4DSQ Somatization	1.10	1.03, 1.17	0.006

^a ^the odds ratio is associated with one unit of the scale

#### Anxiety

**Figure 3:**
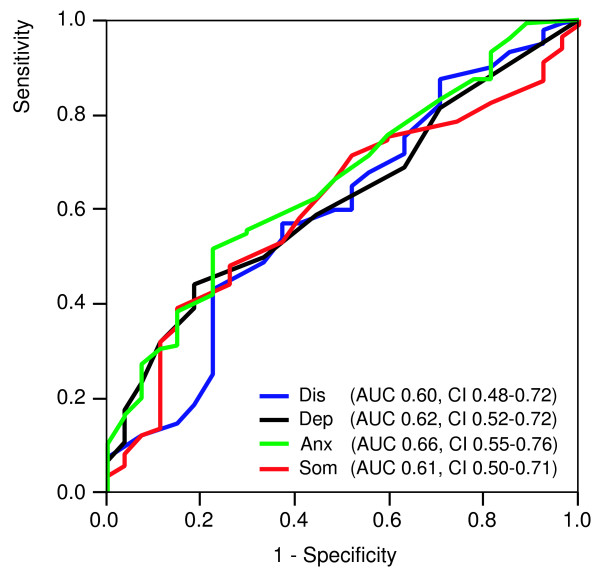
**Receiver operating characteristic (ROC) curves of the 4DSQ scales with respect to detecting any DSM-IV anxiety disorder. **Dis = Distress score, Dep = Depression score, Anx = Anxiety score, Som = Somatization score, AUC = Area Under the Curve, CI = 95% confidence interval.

Table 8 presents the anxiety disorder diagnoses established in study E. One-hundred-seven patients were diagnosed with one anxiety disorder; 72 patients with two or more anxiety disorders. Table 9 shows the mean scores for patients with no, one, and two or more anxiety disorders. Figure 3 shows the ROC curves of the 4DSQ scales to detect any anxiety disorder. The 4DSQ Anxiety scale outperformed the other 4DSQ scales marginally, but not significantly. The AUC of the Anxiety score (0.66) did not significantly differ from the AUCs of the Distress, Depression and Somatization scores (p-values 0.332, 0.490 and 0.401 respectively). A cut-off Anxiety score of ≥ 10 had a sensitivity of 0.62 and a specificity of 0.56. The logistic regression analysis revealed that in the final model only the Anxiety score was a significant predictor of any anxiety disorder, while the other 4DSQ scales did not add any predictive power (Table 10). For every increase of the Anxiety score by 1 point (the scale range is 24 points) the odds for having any (one or more) anxiety disorder(s) increased by 11%.

**Table 8:** Anxiety disorders diagnoses in study E; numbers and percentages

Panic disorder with agoraphobia	81	39%
Panic disorder without agoraphobia	43	21%
Agoraphobia without panic disorder	2	1%
Social phobia	29	14%
Specific phobia	22	11%
Generalised anxiety disorder	78	38%
obsessive-compulsive disorder	10	5%
post-traumatic stress disorder	10	5%
anxiety disorder NOS^a^	3	1%

^a ^NOS = not otherwise specified

**Table 9:** Differences in 4DSQ scores between patients with no, one or two and more anxiety disorders; study E

4DSQ scales	≥ 2 anxiety disorders (n = 72)		1 anxiety disorder (n = 107)		No anxiety disorder (n = 27)		F	df	p
				
	Mean	(SD)	Mean	(SD)	Mean	(SD)			
Distress	24.0	(6.4)	20.4	(6.9)	19.5	(7.5)	7.4	2/203	0.001
Depression	5.3	(3.8)	3.9	(3.8)	2.8	(2.8)	5.5	2/202	0.005
Anxiety	13.3	(6.6)	11.4	(5.7)	8.6	(5.3)	6.3	2/203	0.002
Somatization	18.3	(7.1)	17.0	(6.6)	15.2	(5.7)	2.3	2/202	0.107

**Table 10:** Logistic regression analysis with any anxiety disorder diagnosis as dependent variable and the 4DSQ scores as independent variables; study E

	Odds ratio^a^	95% CI	p
*Initial model*			
4DSQ Distress	0.97	0.88, 1.06	0.440
4DSQ Depression	1.11	0.92, 1.34	0.277
4DSQ Anxiety	1.09	0.99, 1.21	0.088
4DSQ Somatization	1.01	0.94, 1.09	0.726
*Final model*			
4DSQ Anxiety	1.11	1.03, 1.20	0.007

^a ^the odds ratio is associated with one unit of the scale

#### Somatization

**Figure 4:**
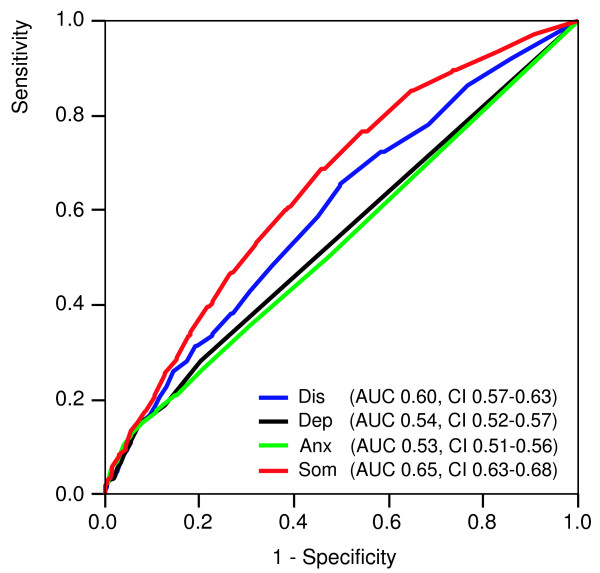
**Receiver operating characteristic (ROC) curves of the 4DSQ scales with respect to detecting suspected (possible or definitive) somatization in general practice patients. **Dis = Distress score, Dep = Depression score, Anx = Anxiety score, Som = Somatization score, AUC = Area Under the Curve, CI = 95% confidence interval.

In study A 1,620 patients had a somatic diagnosis/reason for encounter. Of these patients 773 patients (48%) were suspected by their GP of definite (n = 256) or possible somatization (n = 517). Table 11 shows that all 4DSQ scores paralleled the GPs' level of somatization suspicion. The ROC analysis revealed that the Somatization score was the best discriminator between patients whose GPs suspected (possible or definitive) somatization and those whose GPs did not (Figure 4), the AUC of the Somatization score (0.65) being significantly larger than the AUCs of the Distress, Depression and Anxiety scores (all p-values < 0.001). A cut-off score of ≥ 7 had a sensitivity of 0.60 and a specificity of 0.62 for the GPs' suspicion of somatization in patients with a somatic diagnosis/reason for encounter. The logistic regression analysis revealed that the suspicion of a psychosocial background in patients with somatic symptoms (i.e. somatization) was primarily predicted by the Somatization score (Table 12). With every increase of the Somatization score by 1 point (the scale range is 32 points), the odds to have the GP suspect a (possible or definite) somatization increased by 10%. Furthermore, the Distress score added a little to the Somatization score in predicting the GPs' suspicion of definite somatization.

**Table 11:** Differences in 4DSQ scores in patients with a somatic diagnosis between patients with definite, possible and no somatization according to the GP; study A

4DSQ scales	Definite somatization (n = 256)		Possible somatization (n = 517)		No somatization (n = 847)		F	df	p
				
	Mean	(SD)	Mean	(SD)	Mean	(SD)			
Distress	10.8	(8.0)	8.3	(7.5)	6.7	(6.6)	34.7	2/1617	< 0.001
Depression	1.5	(2.8)	0.9	(2.2)	0.6	(1.7)	18.5	2/1617	< 0.001
Anxiety	2.8	(4.0)	2.0	(3.7)	1.5	(2.6)	16.5	2/1617	< 0.001
Somatization	10.4	(5.9)	8.5	(6.0)	6.3	(5.2)	64.9	2/1617	< 0.001

**Table 12:** Logistic regression analysis with GP's suspicion of somatization in patients with a somatic diagnosis as dependent variable and the 4DSQ scores as independent variables, according to the level of GP's suspicion; study A

	Definite somatization			Possible somatization		
	
	Odds ratio^a^	95% CI	p	Odds ratio^a^	95% CI	p
*Initial model*			
Distress	1.03	1.00, 1.06	0.094	1.01	0.99, 1.03	0.442
Depression	1.04	0.96, 1.12	0.346	1.02	0.96, 1.10	0.521
Anxiety	0.97	0.93, 1.03	0.315	0.98	0.93, 1.02	0.253
Somatization	1.07	1.04, 1.10	< 0.001	1.09	1.07, 1.12	< 0.001
*Final model*						
Distress	1.03	1.00, 1.05	0.012			
Somatization	1.07	1.04, 1.10	< 0.001	1.10	1.08, 1.12	< 0.001

^a ^the odds ratio is associated with one unit of the scale

### Inter-correlations of the 4DSQ scales

**Figure 5:**
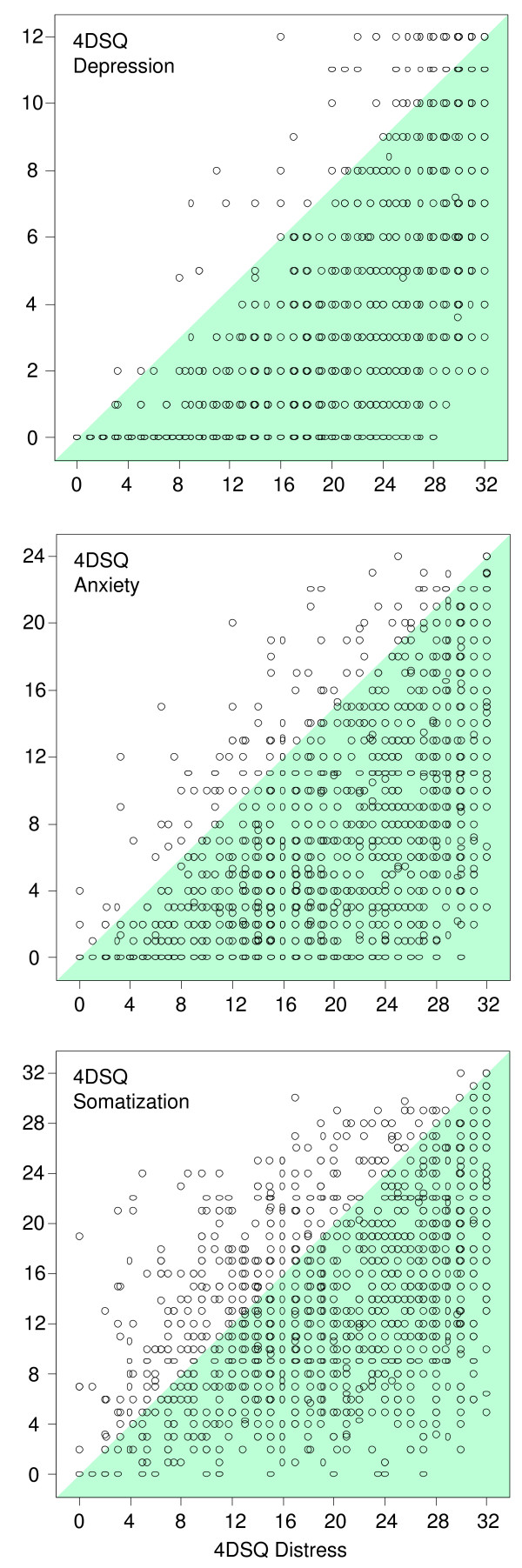
**Scatterplots of the associations between the 4DSQ Distress score and the 4DSQ Depression, Anxiety and Somatization scores. **One dot represents one or more observations (subjects). In the upper graph 94% of the observations are located in the right-lower triangle. The same is true for 93% of the observations in the middle graph, and 79% of the observations in the lower graph.

Table 13 shows the Pearson correlations between the 4DSQ scales. The Distress scale had the highest correlations with the other scales. Table 14 presents the results of the regression analyses. Apparently, the Distress scale was correlated most with the Depression scale and had the lowest proportion of unique variance (about 30%), which was consistent throughout the different samples. Inspection of the scatterplots revealed a peculiar non-reciprocal relationship between Depression and Distress, and between Anxiety and Distress (Figure 5). Elevated scores on Depression or Anxiety were virtually always accompanied by elevated Distress scores, whereas the reverse was not true. The same phenomenon, but to a lesser degree, was visible in the scatterplot of Somatization and Distress. Most patients turned out to be located in the right-lower triangle of the scatterplots: 94% of the patients in the Depression/Distress plot, 93% of the patients in the Anxiety/Distress plot, and 79% of the patients in the Somatization/Distress plot. The scatterplots suggest that Distress "underlied" Depression, Anxiety, and to a lesser extent Somatization.

**Table 13:** Correlations between the 4DSQ scales; Pearson correlation coefficients r

	Depression	Anxiety	Somatization
*Distress*			
- study A	0.70	0.69	0.61
- study B	0.67	0.64	0.59
- studies C-J	0.71	0.56	0.46
*Depression*			
- study A		0.62	0.43
- study B		0.57	0.39
- studies C-J		0.53	0.35
*Anxiety*			
- study A			0.55
- study B			0.50
- studies C-J			0.56

**Table 14:** Proportions shared and unique variance of the 4DSQ scales; standardised Beta-coefficients and explained (shared) variance R^2 ^(results from multiple regression analysis)

	Cronbach's α	Standardised Beta-coefficients				R^2^	Unique variance^a^
				
		Distress	Depression	Anxiety	Somatization		
*Distress*							
- study A	0.93		0.40	0.28	0.29	0.65	0.28
- study B	0.90		0.40	0.26	0.31	0.61	0.29
- studies C-J	0.90		0.55	0.18	0.17	0.57	0.33
*Depression*							
- study A	0.92	0.54		0.29	-0.06	0.53	0.39
- study B	0.82	0.53		0.26	-0.05	0.48	0.34
- studies C-J	0.89	0.61		0.21	-0.05	0.53	0.36
*Anxiety*							
- study A	0.87	0.37	0.28		0.20	0.54	0.33
- study B	0.79	0.36	0.26		0.19	0.47	0.32
- studies C-J	0.88	0.23	0.24		0.37	0.46	0.42
*Somatization*							
- study A	0.83	0.49	-0.07	0.25		0.41	0.42
- study B	0.80	0.49	-0.06	0.22		0.37	0.43
- studies C-J	0.84	0.26	-0.07	0.44		0.34	0.50

^a ^Unique variance = Cronbach's α minus R^2^

### Factorial structure of the 4DSQ

**Figure 6:**
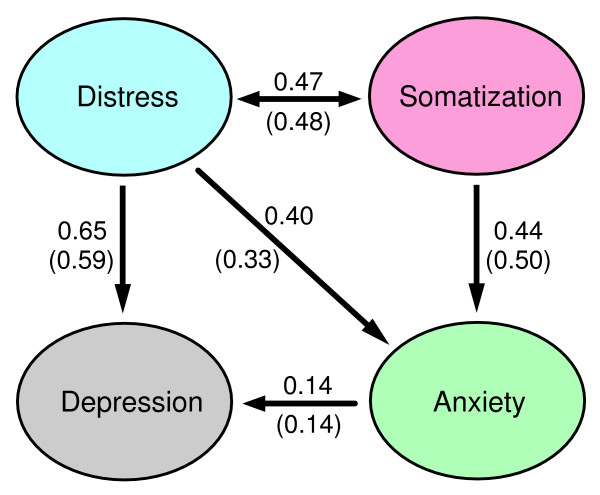
**Diagram of the four-factor model of the 4DSQ. **Numbers are standardised coefficients in the test set (and the exploration set respectively).

In the exploration set the best fitting 4-factor model, with the factors being in accordance with the 4DSQ scales, had a CFI of 0.93. Replication of this model in the test set yielded a CFI of 0.92. Modifications to this model such as omitting an item with a relatively low factor loading (item 3) or allowing five Distress items to cross-load on the Depression factor (items 29, 31, 32, 36 and 37) did not improve the model fit. A 5-factor model treating the five cross-loading Distress items as a fifth factor (representing demoralisation and feelings of impotence), turned out not to improve the model fit either. Moreover, the 5-factor model could not be replicated in the test set. Apparently, the 4-factor model was the most appropriate model. Figure 6 presents the model with the standardised coefficients. The factor loadings in the test set data are shown in Table 1. Note that, in accordance with our conceptual four-dimensional model, Distress was associated with Depression, Anxiety and Somatization. In addition, Somatization was associated with Anxiety but not directly with Depression. The direct association between Anxiety and Depression was relatively small and the simple correlation between Depression and Anxiety (r = 0.53, see Table 13) was apparently largely due to a "common cause" effect of Distress.

### Correlations with other symptom questionnaires

Table 15 presents the Pearson correlations between the 4DSQ scales and the other questionnaires. As expected, all correlations were positive, and almost all were statistically significant. As an example, the General Health Questionnaire (GHQ) showed significant correlations with three 4DSQ scales: Distress, Depression and Anxiety. Does that imply that those three scales independently contributed to the "explanation" of the GHQ score? Table 16 provides the answer: the GHQ score was only associated with the 4DSQ Distress scale, whereas the other 4DSQ scales did not have any independent contributions to the "explanation" of the GHQ score. The correlations between the GHQ and the 4DSQ Depression and Anxiety scales in Table 15 were entirely based on the correlations between the 4DSQ scales themselves. Thirty-four percent of the variance of the GHQ (R^2^) was explained by the 4DSQ scales (almost exclusively by the Distress scale), which was 39% (0.34/0.87) of its reliable variance (i.e. Cronbach's α). Thus, the 4DSQ scales covered only 39% of the error-free variance of the GHQ.

**Table 15:** Correlations between the 4DSQ scales and other symptom questionnaires measuring distress, depression, anxiety and somatization; Pearson correlation coefficients r

	Study	4DSQ scales			
		
		Distress	Depression	Anxiety	Somatization
*Distress*					
- General Health Questionnaire (GHQ)	D	0.58***	0.46***	0.29*	0.12
- Maastricht Questionnaire (MV)	D	0.76***	0.59***	0.46***	0.29*
*Depression*					
- Hospital Anxiety Depression Scale – Depression scale (HADS-Dep)	D	0.67***	0.49***	0.21	0.22
- Zung's Self-Rating Depression Scale (SDS)	D	0.69***	0.59***	0.40**	0.40**
- Symptom Checklist (SCL-90) – Depression	C	0.76***	0.72***	0.53***	0.37***
- Beck Depression Inventory (BDI)	F	0.68***	0.71***	0.63***	0.49***
*Anxiety*					
- State Trait Anxiety Inventory (STAI) – State	H	0.63***	0.55***	0.52***	0.40***
- Hospital Anxiety Depression Scale – Anxiety scale (HADS-Anx)	D	0.58***	0.37**	0.72***	0.35**
- Symptom Checklist (SCL-90) – Anxiety	C	0.57***	0.48***	0.81***	0.56***
- Symptom Checklist (SCL-90) – Agoraphobia	C	0.36***	0.30***	0.80***	0.43***
- Beck Anxiety Inventory (BAI)	F	0.66***	0.53***	0.81***	0.77***
*Somatization*					
- Symptom Checklist (SCL-90) – Somatization	C	0.52***	0.33***	0.56***	0.82***

* p < 0.05, ** p < 0.01, *** p < 0.001 (1-tailed)

**Table 16:** Coverage of the symptom questionnaires measuring distress, depression, anxiety and somatization by the 4DSQ scales; standardised Beta-coefficients and explained (shared) variance R^2 ^(results from linear regression analysis)

	Cronbach's α	Standardised Beta-coefficients				R^2^	Coverage^a^
				
		Distress	Depression	Anxiety	Somatization		
*Distress*							
- GHQ	0.87	0.53*	0.06	0.03	-0.05	0.34	0.39
- MQ	0.88	0.61***	0.13	0.08	0.08	0.60	0.68
*Depression*							
- HADS-Dep	0.76	0.71***	0.02	-0.16	0.10	0.42	0.55
- SDS	0.75	0.42*	0.22	0.08	0.24*	0.54	0.72
- SCL-90-Dep	0.91	0.48***	0.32***	0.17***	-0.04	0.66	0.73
- BDI	0.86	0.25**	0.43***	0.20*	0.02	0.61	0.71
*Anxiety*							
- STAI-State	0.93	0.39***	0.15*	0.16**	0.05	0.43	0.46
- HADS-Anx	0.66	0.30*	-0.01	0.55***	0.10	0.60	0.91
- SCL-90-Anx	0.85	0.15**	0.04	0.64***	0.15***	0.71	0.84
- SCL-90-Ago	0.87	-0.01	-0.10	0.82***	0.05	0.64	0.74
- BAI	0.92	0.14*	0.04	0.46***	0.35***	0.74	0.80
*Somatization*							
- SCL-90-Som	0.82	0.14**	-0.07	0.14**	0.71***	0.72	0.88

* p < 0.05, ** p < 0.01, *** p < 0.001 (1-tailed)

^a ^Coverage = R^2 ^/Cronbach's α

The 4DSQ Distress scale was associated with most comparison questionnaires to some extent (except the SCL-90 Agoraphobia scale), and it was the 4DSQ scale with the highest associations for the GHQ, the MQ, the HADS Depression scale, the SDS, the SCL-90 Depression scale, and the STAI State scale. This indicated that some depression and anxiety inventories measured a lot of distress in 4DSQ terms.

The 4DSQ Depression scale was significantly associated with the BDI, the SCL-90 Depression scale, and the STAI State scale, and it was the 4DSQ scale with the highest associations with the BDI.

The 4DSQ Anxiety scale was associated with all anxiety inventories, as well as with the BDI and the SCL-90 Depression and Somatization scales, and it was the 4DSQ scale with the highest associations with the SCL-90 Agoraphobia and Anxiety scales, the HADS Anxiety scale, and the BAI.

The 4DSQ Somatization scale was associated with the SCL-90 Somatization scale and some of the anxiety and depression inventories. It was the 4DSQ scale with the highest association only with the SCL-90 Somatization scale.

### Associations with stress, personality, and social functioning

Table 17 shows that the associations of life events and work stress with the 4DSQ dimensions were relatively small, and mainly related to Distress. The associations between personality and the 4DSQ scores were quite large, considering the R^2 ^values between 0.25 and 0.65. The associations of the various personality measures with the different 4DSQ dimensions varied.

**Table 17:** Relationships of stress-related measures, personality and social functioning with the 4DSQ scales; standardised Beta-coefficients and explained variance R^2 ^(results from multiple regression analysis)

	Study	Standardised Beta coefficients				R^2^
			
		Distress	Depression	Anxiety	Somatization	
*Stress-related measures*						
- Life events	A	0.18*	-0.12	0.04	0.09	0.04
- Life events	G	0.31	0.07	-0.24	0.10	0.11
- Psychosocial problems	A	0.47***	0.13*	0.08	0.04	0.42
- Psychosocial problems	G	0.35*	0.08	0.04	0.09	0.24
- Psychological job demands (JCQ)	B	0.26***	-0.09***	-0.04	0.03	0.05
- Decision latitude (JCQ)	B	-0.12***	-0.02	-0.05*	-0.07**	0.04
- Social support (JCQ)	B	-0.20***	-0.02	0.01	-0.03	0.05
*Personality*						
- Neuroticism (Ormel)	A	0.38***	0.20***	0.10*	0.22***	0.57
- Neuroticism (Ormel)	G	0.39**	0.23*	0.04	0.31**	0.65
- Neuroticism (NEO-FFI)	F	0.16	0.23*	0.20*	0.10	0.33
- Mastery	B	-0.35***	-0.12***	-0.05**	-0.05*	0.25
- Mastery	C	-0.33***	-0.27***	-0.07	-0.08	0.32
- STAI-trait	H	0.56***	-0.08	0.23***	0.06	0.44
*Social functioning*						
- SF-36 PCS ^a^	F	0.06	-0.07	0.07	-0.41***	0.13
- SF-36 MCS^b^	F	-0.51***	-0.05	-0.21*	0.13	0.39
- Social functioning (5 items)	A	-0.40***	-0.05	0.02	-0.21***	0.33
- Social functioning (5 items)	G	-0.50**	-0.05	0.06	-0.22	0.41
- Sick leave ^c^	A	1.07**	0.91	1.00	1.04	0.12^d^
- Sick leave ^c^	G	1.26**	0.67	1.12	1.14	0.46^d^

* p < 0.05, ** p < 0.01, *** p < 0.001 (2-tailed)

^a ^Physical Component Score

^b ^Mental Component Score

^c ^logistic regression: Odds Ratios in stead of Standardised Beta coefficients

^d ^R^2 ^(Nagelkerke)

Social functioning was mainly associated with Distress and Somatization. The physical component score of the SF-36 was associated with the Somatization score, but the association was relatively small. Mental functioning (SF-36) and social functioning in general was mainly associated with Distress. Sick leave was primarily associated with the 4DSQ Distress score.

## Discussion

### Summary of main findings

#### Distress

The notion that distress is the most general expression of psychological problems of any kind was confirmed by the Distress score, which showed substantial correlations with the scores of various other questionnaires measuring a range of symptoms from distress to depression and from anxiety to somatization. Furthermore, the Distress score was found to be associated with psychosocial stressors, and especially with psychosocial problems such as marital and financial problems and excessive occupational demands. These findings support the assumption that the Distress score is a non-specific indicator of any psychological problem. The Distress score was also shown to be the most important predictor of social dysfunctioning and sick leave. Of special interest is the association between the Distress score and any psychosocial diagnosis established by the GP. Considering that such diagnoses require consensus between GPs and patients on the psychosocial nature of the symptoms, it is plausible that distress plays a role in motivating patients to seek help and to discuss psychological issues with their doctor. Indeed, one of the practical applications of the 4DSQ in general practice is to increase patients' awareness of their distress, and to encourage their acknowledgement of psychological problems and their readiness to discuss these problems with their doctor.

#### Depression

The 4DSQ Depression scale was found to correlate with other depression scales, particularly with the BDI, which focuses on depressive cognitions [49]. After adjusting for the 4DSQ Distress score, the 4DSQ Depression scale showed little correlation with some other depression scales such as the HADS Depression scale and the SDS of Zung, which appeared to be more closely associated with the 4DSQ Distress score. These findings may indicate that the 4DSQ Depression scale measures more severe depressive disorders, whereas the Distress scale measures milder depressive disorders. Indeed, we found that the 4DSQ Depression and Distress scores performed almost equally well in detecting the whole range (from mild to severe) of DSM-IV major depressive disorders. However, the Distress score did not add any predictive power to the Depression score in the logistic regression analysis. Therefore, the 4DSQ Depression score seems to be sufficient to detect depressive disorders. Considering that the Depression score reflects the likelihood of the presence of a DSM-IV depressive disorder, we recommend two cut-off points in clinical practice: a relatively low cut-off point with high sensitivity to exclude a depressive disorder, and a relatively high cut-off point with high specificity to identify a high likelihood of depressive disorder. For the GP, a Depression score between the two cut-off points is a "prompt to consider" the presence of a depressive disorder [50]. Then, depending on the circumstances and the patient's history, the doctor might decide to wait and see, and to re-evaluate the patient a few weeks later, or to continue with a psychiatric diagnostic interview. However, a Depression score above the higher cut-off point is a "prompt to act" [50], i.e. to diagnose a depressive disorder without delay.

#### Anxiety

The 4DSQ Anxiety scale was found to correlate well with other scales that are used to measure anxiety, including the SCL Agoraphobia and Anxiety scales, the BAI, and the HADS Anxiety scale. However, one other anxiety scale, the STAI State scale, was not found to correlate much with the 4DSQ Anxiety scale, but appeared to correlate more with the Distress scale. On further consideration, the anxiety concept measured by the STAI State scale seems to be quite different from the anxiety concept measured by the other anxiety scales, in that it seems to be related to "normal" nervousness and lack of wellbeing (i.e. distress), as opposed to the kind of "abnormal" anxiety that is expressed in panic attacks and phobic anxiety. It is this "abnormal" anxiety, which is characteristic of anxiety disorders, that is measured by the 4DSQ Anxiety scale. Yet, we failed to demonstrate unequivocal criterion validity of the Anxiety scale with respect to standardised DSM-IV anxiety disorder diagnoses. In retrospect, we suspect that study E was not ideal for investigating the criterion validity of the Anxiety scale because the patients were highly selected (for anxiety), leaving little contrast between patients with and without an anxiety disorder. More research, with more heterogeneous study samples, is needed to establish the criterion validity of the 4DSQ Anxiety scale.

#### Somatization

The 4DSQ Somatization scale was shown to measure exactly the same construct as the SCL Somatization scale (taking the reliability of both scales into account). Furthermore, we demonstrated criterion validity with respect to the GP's suspicion of somatization in patients with a somatic diagnosis/reason for encounter. Although the relatively low AUC (0.65) indicates that the Somatization score is not a perfect predictor of a GP's suspicion of somatization, the association between the score and the GP's assessment is interesting. Apparently, GPs recognise something that is expressed through an elevated Somatization score in a patient. It should be realised at this point that the GPs and patients probably were not able to discuss any psychosocial issues, because otherwise the GPs would have established a psychosocial diagnosis. Thus, the GP's assessment of the presence of a psychosocial background in patients with somatic symptoms must have been the result of a rather subjective process with questionable reliability. Assuming a relatively low reliability of our "criterion" for somatization, and considering that validity can never surpass reliability, an AUC-value of 0.65 may not be too bad at all. One of the practical applications of the 4DSQ in general practice is to obtain Somatization scores for patients who the GP suspects of having complaints with a psychological background. Discussing the 4DSQ scores with the patient provides valuable opportunities to address any psychosocial issues.

#### Relationships between the 4DSQ dimensions

We found a special relationship between Distress, on the one hand, and Depression and Anxiety (and Somatization to a lesser extent) on the other hand: in most patients Distress seemed to underlie Depression and Anxiety (and Somatization to a lesser extent). To a large extent this peculiar relationship between the 4DSQ dimensions is responsible for the high correlations between Distress, on the one hand, and Depression, Anxiety and Somatization on the other hand. A low Distress score is highly predictive of the Depression and Anxiety scores (which must be low), but a high Distress score is minimally predictive of the Depression and Anxiety scores (which can be anywhere between low and high). This special relationship was also evident in the factorial structure of the 4DSQ scales. These findings support the use of the 4DSQ Distress scale as a screener for psychological problems of any kind in primary care.

It seems plausible to attach causal inferences to the relationships between the 4DSQ dimensions in accordance with our conceptual four-dimensional model [51]. Whenever individuals are confronted with the pressure of stress in their life or work, they start to experience distress because they have to put more effort into coping with the stressors, trying to maintain their normal level of social functioning. This is the most basic, most general, most "normal" response to stress. However, some of these people will also experience bodily symptoms (somatization) to some degree, due to a certain tendency [12]. Moreover, distress may act as a steppingstone to the development of a depressive and/or anxiety disorder in individuals with specific vulnerabilities. Of course, longitudinal studies are needed to confirm this interpretation of our cross-sectional data.

### Limitations and strengths

An important limitation of this study has to do with the fact that some of the data we used were primarily collected for other purposes. Furthermore, all data were cross-sectional. The ultimate test of clinical validity requires prediction of relevant events in a longitudinal design. Strengths of the study include the amount of data analysed, and the fact that many results have been confirmed in different samples.

## Conclusion

The Four-Dimensional Symptom Questionnaire (4DSQ) appears to be a valid self-report questionnaire to measure distress, depression, anxiety and somatization in primary care.

## Abbreviations

4DSQ: Four-Dimensional Symptom Questionnaire

ANOVA: analysis of variance

AUC: area under de ROC curve

BAI: Beck Anxiety Inventory

BDI: Beck Depression Inventory

CFA: confirmatory factor analysis

CFI: comparative fit index

DSM: Diagnostic and Statistical Manual of mental disorders

GHQ: General Health Questionnaire

GP: general practitioner

HADS: Hospital Anxiety and Depression Scale

JCQ: Job Content Questionnaire

MQ: Maastricht Questionnaire

NEO-FFI: NEO Five Factor Inventory

NB: nervous breakdown

ROC: receiver operating characteristic

SCID: Structured Clinical Interview for DSM-IV

SCL: Symptom Checklist

SF-36: Short Form Health Survey

SDI: Short Depression Interview

SDS: Self-rating Depression Scale of Zung

STAI: State-Trait Anxiety Inventory

## Appendix 1

### Description of the studies from which the datasets have been used in this paper

*Study A *aimed to investigate the characteristics of patients with a nervous breakdown (NB) in general practice [4]. Thirty-seven GPs handed out a questionnaire containing the 4DSQ to a random sample of 3,495 patients aged 15 to 64, and registered their diagnoses or reasons for encounter. The GPs recorded also their assessment of the somatic or psychological background of the consultation on a 5-point scale, from "exclusively somatic" (score 1) to "exclusively psychosocial" (score 5). The questionnaire was returned by 2,127 patients (response rate 61%).

From the respondents a stratified sample (n = 612) was selected for further investigation: all patients with a psychiatric diagnosis as registered by the GP (n = 85), all patients with a NB diagnosis (n = 141), and a sample of patients with psychological or somatic symptoms (n = 386). The follow-up study included a postal questionnaire, issued 1–2 weeks later, containing a checklist of psychosocial problems [42], a life events checklist, a neuroticism questionnaire [45], a 5-item social disability questionnaire, and a single question about current sick leave from work. The second questionnaire was returned by 458 patients after a mean interval between the questionnaires of 12.0 days (SD 5.5).

*Study B *was part of an occupational health survey [52]. All employees of a telecom company (n = 7,522) received a questionnaire containing the 4DSQ, a job stress questionnaire, the Job Content Questionnaire (JCQ) [43], and a mastery questionnaire, the Pearlin Mastery Scale [47]. The questionnaire was returned by 3,852 employees (response rate 51%).

*Study C *was designed to investigate an occupational health care intervention for employees who were on sick leave because of an adjustment disorder [53]. There had to be a recent identifiable stressor and the patient had to have at least 8 out of 17 distress symptoms. Patients were excluded if one of the DSM-IV exclusion criteria for adjustment disorder held (e.g. the patient had a major depression). The physicians included 280 patients. The baseline assessment included the 4DSQ, the Symptom Checklist (SCL-90) [31,32], as well as the Pearlin Mastery Scale [47].

*Study D *was designed to investigate the reliability and validity of the Short Depression Interview (SDI) to assess depressive symptoms and to diagnose major depression [22]. Fourteen GPs selected 55 patients (aged 15 years and older) with a psychological problem of any kind, and at least 3 out of the following 6 symptoms: fatigue, sleeping problems, nervousness, irritability, feeling depressed, feeling anxious. The GPs interviewed the patients twice, a few days apart. After the first interview, the patients filled in the 4DSQ, the Self-rating Depression Scale of Zung (SDS) [35,36] and the General Health Questionnaire (GHQ) [27,28]. After the second interview, the patients filled in the 4DSQ, the Hospital Anxiety and Depression Scale (HADS) [33,34] and the Maastricht Questionnaire (MQ) [29], which assessed "vital exhaustion" [30]. According to the SDI more than half of the patients fulfilled criteria of DSM-IV major depression.

*Study E *was designed to compare the effectiveness of three interventions for panic disorder and generalised anxiety disorder in primary care patients [54]. Forty-six GPs identified 258 adult patients, aged 18 years and older, who scored 5 or more on the Short and Simple Screening Interview [55]. Reasons for exclusion were: the presence of an organic mental disorder, mental retardation or a psychotic disorder, treatment of an anxiety disorder in the recent past, use of antidepressants or the use of a benzodiazepine in daily doses of more than 30 mg oxazepam equivalents. The patients were given the 4DSQ and a clinical assessment by a mental health professional using the Structured Clinical Interview for DSM-IV (SCID) [23]. Two-hundred-and-thirty-seven patients completed the 4DSQ; 206 of them completed the SCID interview as well. According to this interview, 87% of the patients fulfilled criteria of one or more DSM-IV anxiety disorders.

*Study F *was designed to investigate the effectiveness of an antidepressant in primary care patients with minor or mild-major depression. Fifty-three GPs included 181 patients, aged 18 years and older, with 3 to 6 depressive symptoms mentioned in the DSM-IV criteria for major depression. Patients with severe suicidal behaviour, addiction, apparent cognitive decline (dementia), or psychotic symptoms, were excluded. At baseline, the patients filled in the 4DSQ, the Beck Depression Inventory (BDI) [37,38], the Beck Anxiety Inventory (BAI) [39], the NEO-FFI Neuroticism scale [46,56]**, **and the Short Form Health Survey (SF-36) [57,58]. Three patients did not fill in the questionnaires.

*Study G *was a parallel study of study A. Nineteen primary care based social workers handed out the 4DSQ to a sample of their clients (n = 83). Seventy-seven clients returned the questionnaire (response rate 93%). All respondents filled in the second questionnaire inquiring after psychosocial problems, life events, neuroticism, social disability, and sick leave, after a mean interval of 8.3 (SD 9.8) days.

*Study H *was designed to test the reliability and validity of the 4DSQ in physiotherapy patients [59]. Twenty-three primary care based physiotherapists handed out the 4DSQ and the State-Trait Anxiety Inventory (STAI) [40,41] to a consecutive sample of new patients. The questionnaires were returned by 382 patients.

*Study I *aimed to assess stability and change of the 4DSQ in general practice patients over a 1–2 week period. Five GPs handed out the 4DSQ to 98 patients with various psychological problems. The questionnaire was returned by 86 patients (response rate 88%). One week later, a second 4DSQ was sent to these patients (response: n = 66, response rate 77%). The second 4DSQ was accompanied by one question about the patients' perception of stability/change in their symptoms. This global impression (GI) question had 5 response options: "improved definitely" (score 1), "improved somewhat" (score 2), "unchanged" (score 3), "deteriorated somewhat" (score 4), and "deteriorated definitely" (score 5).

*Study J *aimed to estimate the amount of time it takes for general practice patients to fill in the 4DSQ. In one general practice (5 GPs) two extra questions were added temporarily to the 4DSQ that was used routinely in the practice. The questionnaire was filled in by 129 patients.

## Competing interests

BT is the copyright owner of the 4DSQ and receives copyright fees from companies that use the 4DSQ on a commercial basis (the 4DSQ is freely available for non-commercial use in health care and research). BT received fees from various institutions for workshops on the application of the 4DSQ in primary care settings.

## Authors' contributions

BT performed the statistical analyses (except for the confirmatory factor analysis) and drafted the manuscript. HWJvM, BWJHP and WABS conceived the idea for the manuscript. HJA performed the confirmatory factor analysis. HCWdV assisted in the analyses of reliability, precision, smallest detectable change and responsiveness. MLMH acquired the data of study F and assisted in the analyses of these data. CAvB acquired the data of study E. CAvB and AJLMvB assisted in the analyses of the data of study E. JJLvdK acquired the data of study C and assisted in the analyses of these data. HWJvM, HCWdV, HJA, BWJHP, AJLMvB and WABS helped to draft the manuscript. All authors read and approved the final manuscript.

## Pre-publication history

The pre-publication history for this paper can be accessed here:



## Supplementary Material

Additional file 1 Reliability, precision and smallest detectable change. Paper assessing the reliability, precision and smallest detectable change of the 4DSQ scales, using the data of studies C through J. Click here for file

Additional file 2 Responsiveness. Paper assessing the responsiveness of the 4DSQ scales, using the data of study I. Click here for file

Additional file 3 Respondent burden. Paper assessing the time needed to fill in the 4DSQ, using the data of study J. Click here for file
