# Supplementary material for: The Four-Dimensional Symptom Questionnaire (4DSQ): a validation study of a multidimensional self-report questionnaire to assess distress, depression, anxiety and somatization
Source: BMC Psychiatry. 2006 Aug 22;6:34. doi: 10.1186/1471-244X-6-34 (PMC1590008; doi:10.1186/1471-244X-6-34)
Supplement: Additional file 1 — Reliability, precision and smallest detectable change. Paper assessing the reliability, precision and smallest detectable change of the 4DSQ scales, using the data of studies C through J. [file 1471-244X-6-34-S1.pdf]

Additional file 1

Reliability, precision and smallest detectable change

The reliability of a test refers to the proportion of the total variance that is error-free. In clinical practice our interest usually concerns a patient's "true score" on the variable that we are trying to measure. The observed score is an estimate of this unmeasurable true score, and the question is how far the observed score can be "off the mark". This can be calculated using the standard error of measurement (SEM).[1] When we are trying to evaluate change scores, then we are dealing with two measurements, each with a measurement error. The question is how big a change should be before we can confidently conclude that there is a real change instead of chance fluctuation. This can be calculated using the standard error of estimate (SEE).[1]

Methods

We used the data of studies C through J pooled together (total n = 1,424). As an estimate of the reliability of the 4DSQ scales we calculated the internal consistency coefficient Cronbach's α [1]. The standard error of measurement (SEM), being the standard deviation of infinite repeated measurements (under the same condition) around their mean, and thus the standard deviation of infinite repeated measurements around the "true score", can be used to determine how far one measurement can be off the mark as an estimate of the "true score" [1]. We calculated the SEM from the standard deviation and the α coefficient of the scores, using the formula: $SEM = SD*\sqrt{(1-\alpha)}$, in which SD is the standard deviation and α is the reliability coefficient [2]. The interval between the observed score minus 1.96*SEM and the observed score plus 1.96*SEM can be interpreted as the 95% confidence interval for the "true score". In other words, the observed score can be 1.96*SEM far off the mark; this we call the measurement precision. The precision as a percentage of the range of the scale is denoted the precision index.

The standard deviation of the differences between infinite repeated measures (under the same condition) is denoted the standard error of estimate (SEE) [2]. This value can be used to estimate the probability that the difference between two repeated measures is due to measurement error. We calculated the standard error of estimate (SEE) from the SEM, using the formula: $SEE = SEM*\sqrt{2}$ [2]. In the case of two repeated measurements under unchanged conditions the second test score can, with 95% confidence, be expected to lie between the observed score minus 1.96*SEE and the observed score plus 1.96*SEE. Under unchanged conditions 95% of the differences between two scores do not exceed 1.96*SEE. This measure is called the smallest detectable change (SDC) [3], the smallest difference between two repeated measures that can, with 95% confidence, be ascribed to a real change in the variable measured [4]. The SDC as a percentage of the scale range we denoted the SDC index.

Results

Table A1.1 presents Cronbach's α coefficients, the measurement precision and the smallest detectable changes (SDC) of the 4DSQ scales. As an estimate of a patient's "true score", a single score on one of the 4DSQ scales

could be 15-19% far off the mark (precision index). The SDC was about 21-27% of the scale range for all 4DSQ scales.

Table A1.1: Reliability, precision and smallest detectable change of the 4DSQ-scales; studies C through J pooled

		Distress	Depression	Anxiety	Somatization
- Range scale		32	12	24	32
- Standard deviation (SD)		7.7	3.6	6.2	7.0
- Cronbach's α		0.90	0.89	0.88	0.84
- SEM	[SD*√(1-α)]	2.43	1.19	2.15	2.80
- Precision	[SEM*1.96]	4.76	2.33	4.21	5.49
- Precision index	[precision/range]	15%	19%	18%	17%
- SEE	[SEM*√2]	3.44	1.68	3.04	3.96
- SDC	[SEE*1.96]	6.74	3.29	5.96	7.76
- SDC index	[SDC/range]	21%	27%	25%	24%

SEM = standard error of measurement
SEE = standard error of estimate
SDC = smallest detectable change

Discussion

The reliability of the 4DSQ scales, measured here as the internal consistency, appears to be good with alpha coefficients well above 0.80. As an estimate of a patient's true score, a single score on one of the 4DSQ scales can be 15-19% far off the mark (precision index). As conservative estimates of the SDC's of the 4DSQ scales we adopted the following values: for Distress 7 points, for Depression 4 points, for Anxiety 6 points, and for Somatization 8 points. Only a change score of at least this magnitude can be taken (with 95% confidence) as proof of a real change (i.e. of a change in the "true score").

References

1. Streiner DL, Norman GR: *Health measurement scales. A practical guide to their development and use.* Oxford: Oxford University Press; 1995.

2. Crocker L, Algina J: *Introduction to classical and modern test theory.* Fort Worth: Harcourt Brace Jovanovich College Publishers; 1986.

3. De Vet HCW, Bouter LM, Bezemer PD, Beurskens AJHM: **Reproducibility and responsiveness of evaluative outcome measures. Theoretical considerations illustrated by an empirical example.** *Int J Technol Assess Health Care* 2001, **17:**479-487.

4. Beckerman H, Roebroeck ME, Lankhorst GJ, Becher JG, Bezemer PD, Verbeek ALM: **Smallest real difference, a link between reproducibility and responsiveness.** *Qual Life Res* 2001, **10:**571-578.
