# Supplementary material for: The Four-Dimensional Symptom Questionnaire (4DSQ): a validation study of a multidimensional self-report questionnaire to assess distress, depression, anxiety and somatization
Source: BMC Psychiatry. 2006 Aug 22;6:34. doi: 10.1186/1471-244X-6-34 (PMC1590008; doi:10.1186/1471-244X-6-34)
Supplement: Additional file 2 — Responsiveness. Paper assessing the responsiveness of the 4DSQ scales, using the data of study I. [file 1471-244X-6-34-S2.pdf]

Additional file 2

Responsiveness

Responsiveness refers to the extent to which changes in 4DSQ scores over a specified time frame correspond to changes in a reference measure. We are dealing here with “anchor-based” or “external” responsiveness [1].

Methods

In study I GP patients with psychosocial problems (n = 86) filled in the 4DSQ twice, with a mean interval of 10.0 (SD 2.2) days. The reference measure used was the patients’ perception of change in their symptoms expressed in a 5-point global impression (GI) score.

First, we looked at floor and ceiling effects of the 4DSQ baseline scores. After all, patients at the floor of a scale at baseline cannot show any improvement at a second measurement. Likewise, patients at the ceiling of a scale cannot show any deterioration on that scale. Second, we calculated the correlation (Pearson coefficients) between the change scores of the 4DSQ scales and the GI score. Third, mean change scores (and standard deviations) for the 4DSQ scales were calculated for 3 groups of patients, those improved, those unchanged, and those deteriorated. Fourth, the patients were divided into 2 groups: improved and not improved and receiver operating characteristic (ROC) analyses were performed. The area under the curve (AUC) was calculated as a measure of the ability of the 4DSQ change scores to discriminate between improved and not improved patients [1]. Finally, logistic regression was performed with subjective improvement as dependent variable and the 4DSQ change scores as independent variables, for the 4DSQ scales separately. Odds ratio’s (and 95% confidence intervals [CI]) were calculated. Predicted probabilities for improvement were then plotted against the change score for the most responsive 4DSQ scale.

Results

The GI question was filled in by 59 patients. Twenty patients indicated that they had improved (14 somewhat, and 6 definitely). Thirty-three patients stated they had remained about the same, and six patients indicated their symptoms had deteriorated (5 somewhat, and 1 definitely). The baseline scores of the Depression and Anxiety scales showed substantial floor effects (Table A2.1). The Distress and Somatization change scores showed significant correlations with the subjective GI score. Subjectively improved patients did indeed experience a reduction in mean 4DSQ scores. Strangely enough, subjectively deteriorated patients also experienced a small reduction in 4DSQ scores, except for the Somatization score. However, the number of deteriorated patients was small and the changes were statistically non-significant. The ROC analyses showed that the Distress change score had the best properties for discriminating between subjectively improved en unimproved patients (AUC 0.88). The optimal cut-off point for the Distress change score to “detect” subjective improvement would be ≤ –4 (sensitivity 0.80, specificity 0.77). Figure A2.1 depicts the predicted probability of a patient feeling improved as a function of the Distress change score. When a patient’s Distress score had been reduced by 7 points (the smallest detectable change of the Distress scale) we can be 95% confident that this patient had indeed a genuine distress reduction. However, it is interesting to note from Figure A2.1 that this patient had a 51% probability to

acknowledge this improvement. Some patients failed to recognize a distress reduction that was statistically significant but, apparently, too small to be clinically relevant. Only when the Distress score had been reduced by 17 points 95% of the patients would recognize a reduction of psychological symptoms.

Table A2.1: Responsiveness analysis

	Distress	Depression	Anxiety	Somatization
Baseline, percentage at floor (score 0)	0	39	23	1
Baseline, percentage at ceiling (max. scale score)	5	7	0	1
Correlation with subjective change: r (95% CI)[a]	0.48 (0.25, 0.65)	0.24 (-0.02, 0.47)	0.24 (-0.02, 0.46)	0.30 (0.04, 0.53)
Mean change score (SD) improved (n = 20)	-9.3 (6.1)	-2.6 (3.1)	-3.0 (3.1)	-3.5 (4.4)
Mean change score (SD) unchanged (n = 33)	-0.8 (4.8)	-0.4 (2.2)	-1.0 (4.8)	-0.5 (5.7)
Mean change score (SD) deteriorated (n = 6)	-2.0 (5.3)	-1.3 (2.0)	-0.4 (3.3)	2.2 (4.0)
ROC analysis: AUC (95% CI)[b]	0.88 (0.79, 0.97)	0.71 (0.57, 0.85)	0.67 (0.52, 0.81)	0.69 (0.55, 0.83)
Logistic regression: odds ratio (95% CI)[bc]	0.74 (0.63, 0.87)	0.74 (0.59, 0.94)	0.86 (0.73, 1.02)	0.86 (0.75, 0.99)

[a] Pearson correlation between change scores and subjective change score (5-points scale)

[b] Dependent variable is subjective change dichotomized into improved or not improved

[c] The odds ratio is associated with one point change score per scale

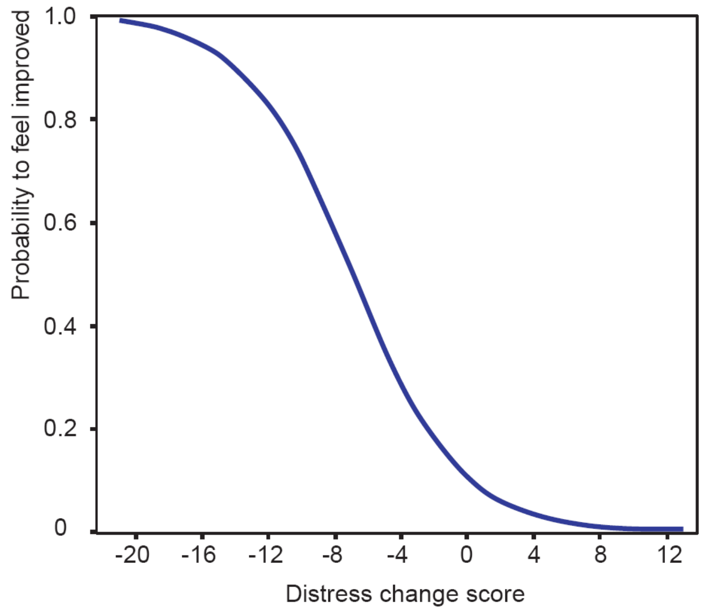

Figure A2.1: Predicted probability of subjective improvement based on the Distress change score

At a Distress change score of -7 points, the patient has a 51% probability to feel subjectively improved.

Discussion

Since the 4DSQ measures four different kinds of symptoms, it is not unreasonable to expect that the 4DSQ scales would differ in their responsiveness to changes in symptoms as perceived by the patients. The 4DSQ Distress scale turned out to be responsive to perceived global improvement. In this respect the Distress scale was clearly superior to the other 4DSQ scales, which may be explained by the heterogeneity of the sample, and the relatively short follow-up of 10 days. We suspect that, when patients are asked to judge any change in their otherwise unspecified symptoms, they tend to focus primarily on their general distress [2]. In other words, when GP-patients with psychological complaints experience subjective improvement within a short period of 10 days, they experience primarily a reduction of distress, rather than depression, anxiety or somatization.

We were not able to investigate the responsiveness of the Depression, Anxiety and Somatization scales because the sample comprised too little patients with high scores on these scales. Furthermore, we were not able to investigate the responsiveness to deterioration of psychological symptoms because most patients in the sample either remained stable or improved. A longer follow-up period and specifically selected samples of patients specifically treated for depression, anxiety disorder or somatization would be required.

References

1. Husted JA, Cook RJ, Farewell VT, Gladman DD: **Methods for assessing responsiveness: a critical review and recommendations.** *J Clin Epidemiol* 2000, **53:**459-468.

2. Clark LA, Watson D: **Tripartite model of anxiety and depression: psychometric evidence and taxonomic implications.** *J Abnorm Psychol* 1991, **100:**316-336.
