# Supplementary material for: The Four-Dimensional Symptom Questionnaire (4DSQ): a validation study of a multidimensional self-report questionnaire to assess distress, depression, anxiety and somatization
Source: BMC Psychiatry. 2006 Aug 22;6:34. doi: 10.1186/1471-244X-6-34 (PMC1590008; doi:10.1186/1471-244X-6-34)
Supplement: Additional file 3 — Respondent burden. Paper assessing the time needed to fill in the 4DSQ, using the data of study J. [file 1471-244X-6-34-S3.pdf]

Additional file 3

Respondent burden

Respondent burden is not a psychometric attribute, but it is an important characteristic of a questionnaire [1]. Here we report on the time respondents need to fill in the 4DSQ.

Methods

Study J was conducted in one general practice with five GPs. Two extra questions were added temporarily to the 4DSQ that was used routinely in the practice. The first question asked the patient to write down the time before answering the 4DSQ items; the second question asked the patient to write down the time after completion of the 4DSQ. The 4DSQ was filled in by 129 patients while 110 of them answered the time registration questions. The distribution of the time spend filling in the 4DSQ was expressed as the median and inter-quartile range. Furthermore, we investigated the influence of gender, age and levels of 4DSQ scores on the fill in time using logistic regression analysis with time as dependent variable, dichotomized at the median value.

Results

The median time spend on filling in the 4DSQ was 7 minutes (inter-quartile range 5-10 minutes). Four patients needed more than 30 minutes, up to one hour, to complete the 4DSQ. However, it is not certain that these patients really needed all that time or that they interrupted filling in the questionnaire to do something else like making a cup of coffee, or answering the phone. Table A3.1 presents the results of the logistic regression analysis to investigate the influence of gender, age and levels of 4DSQ scores on the fill in time. In the multivariate model the Somatization score was associated with a shorter fill in time (odds ratio for a longer fill in time 0.93; 95% CI 0.87, 1.00). However, in a univariate model the Somatization score was not significantly associated with the fill in time (odds ratio = 0.96; CI 0.91, 1.02; p = 0.161). The fill in time was not associated with age.

Discussion

Most patients (75%) are able to fill in the 4DSQ within 10 minutes.

References

1. Scientic Advisory Committee of the Medical Outcomes Trust: **Assessing health status and quality-of-life instruments: attributes and review criteria.** *Qual Life Res* 2002, **11:**193-205.

Table A3.1: Logistic regression analysis with the fill in time above the median (>7 minutes) as dependent variable and gender, age, and the 4DSQ scores as independent variables

	Odds ratio	95% CI	p
Gender (F=0, M=1)	1.22	0.55, 2.74	0.626
Age (years)	1.02	0.98, 1.05	0.322
4DSQ Distress	1.02	0.94, 1.11	0.681
4DSQ Depression	1.04	0.88, 1.23	0.637
4DSQ Anxiety	1.04	0.95, 1.14	0.399
4DSQ Somatization	0.93	0.87, 1.00	0.037
